# Genetic Association between Lipid-Regulating Drug Targets and Diabetic Retinopathy: A Drug Target Mendelian Randomization Study

**DOI:** 10.1155/2024/5324127

**Published:** 2024-05-09

**Authors:** Shengnan Chen, Ming Zhang, Peng Yang, Jianbin Guo, Lin Liu, Zhi Yang, Kai Nan

**Affiliations:** ^1^Department of Joint Surgery, HongHui Hospital, Xi'an Jiaotong University, Xi'an 710054, Shaanxi, China; ^2^Medical Department of Xi'an Jiaotong University, Xi'an, Shaanxi 710048, China; ^3^Department of General Practice, HongHui Hospital, Xi'an Jiao Tong University, Xi'an 710054, Shaanxi, China

## Abstract

**Background:**

Diabetic retinopathy (DR) is a diabetic microvascular complication and a leading cause of vision loss. However, there is a lack of effective strategies to reduce the risk of DR currently. The present study is aimed at assessing the causal effect of lipid-regulating targets on DR risk using a two-sample Mendelian randomization (MR) study.

**Method:**

Genetic variants within or near drug target genes, including eight lipid-regulating targets for LDL-C (HMGCR, PCSK9, and NPC1L1), HDL-C (CETP, SCARB1, and PPARG), and TG (PPARA and LPL), were selected as exposures. The exposure data were obtained from the IEU OpenGWAS project. The outcome dataset related to DR was obtained from the FinnGen research project. Inverse-variance-weighted MR (IVW-MR) was used to calculate the effect estimates by each target. Sensitivity analyses were performed to verify the robustness of the results.

**Results:**

There was suggestive evidence that PCSK9-mediated LDL-C levels were positively associated with DR, with OR (95% CI) of 1.34 (1.02-1.77). No significant association was found between the expression of HMGCR- and NPC1L1-mediated LDL-C levels; CETP-, SCARB1-, and PPARG-mediated HDL-C levels; PPARA- and LPL-mediated TG levels; and DR risk.

**Conclusions:**

This is the first study to reveal a genetically causal relationship between lipid-regulating drug targets and DR risk. PCSK9-mediated LDL-C levels maybe positively associated with DR risk at the genetic level. This study provides suggestive evidence that PCSK9 inhibition may reduce the risk of DR.

## 1. Introduction

Diabetic retinopathy (DR), present in approximately 30% of patients with diabetes, is a common diabetic microvascular complication and a leading cause of vision loss in the adult working population [1–3]. As the prevalence of type 2 diabetes continues to rise globally, the prevalence of DR is expected to increase in parallel [2]. However, there is currently a lack of effective strategies to reduce the risk of DR. Therefore, there is an urgent need to identify some therapeutic targets to delay the onset and progression of DR.

It is known that disturbances in lipid metabolism can induce increased inflammation and oxidative stress [4]. The retina is particularly vulnerable to oxidative damage due to its high metabolic rate and rapid oxygen consumption [5, 6]. Therefore, increased oxidative stress can trigger apoptosis of retinal endothelial cells, pericytes, and ganglion cells [7]. Meanwhile, inflammation and oxidative stress can also lead to endothelial injury and dysfunction in blood vessel walls [8], resulting in macro- or microvascular complications such as retinopathy and nephropathy [9]. Consequently, disorders in lipid metabolism may contribute to the onset and progression of DR. However, inconsistent evidence has been observed regarding the association between several circulating lipoproteins and DR [10]. Some epidemiological studies found no significant association between total cholesterol (TC), high-density lipoprotein cholesterol (HDL-C), and DR development [11, 12]. But other studies observed that triglyceride (TG) and TC levels were significantly associated with DR risk in diabetic patients [13, 14]. These discrepant findings could be attributed to measurement errors, uncontrolled confounders, and reverse causality in epidemiological studies [15], which can induce spurious associations or mask the real risk factors [16].

Mendelian randomization (MR) analysis is a method that uses genetic variants, randomly allocated at conception, as instrumental variables (IVs) to estimate the causal effect of exposure on outcome [17]. It minimizes biases caused by confounding factors and reverse causation [18], thereby facilitating a more robust inference of causal relationships between exposure and outcome. Therefore, MR analysis may be a reliable approach to help elucidate the association between lipid levels and DR risk. While a previous MR meta-analysis did not find clear causal links between lipid levels (including HDL-C, low-density lipoprotein cholesterol (LDL-C), TC, and TG) and DR [19], a recent MR study reported an association between HDL-C levels and DR risk [7]. Consequently, inconsistent evidence also exists regarding the effect of lipids on DR. It is worth noting that the aforementioned studies primarily focused on investigating the influence of lipid concentrations on DR, while neglecting to investigate specific targets. Hence, the specific target for regulating lipid metabolism to modulate the progression of DR remains unknown. Therefore, exploring the association between lipid-regulating drug targets and DR could effectively address this research gap and contribute to identifying potential preventive and therapeutic targets for DR.

Lipid-regulating drugs can be classified according to their predominant targets, including drugs that lower TG and LDL-C, as well as those that raise HDL-C. Common LDL-C lowering targets include HMG-CoA reductase (HMGCR) inhibitors, proprotein convertase subtilisin/kexin type 9 (PCSK9) inhibitors, and Niemann–Pick C1-like 1 (NPC1L1) inhibitors [20]. The most commonly used TG-lowering drug, fenofibrate, primarily targets peroxisome proliferator-activated receptor alpha (PPARA) [20]. Lipoprotein lipase (LPL) is also a therapeutic target in modulating TG levels [21, 22]. Targets are aimed at increasing HDL-C levels including cholesterol ester transfer protein (CETP), scavenger receptor class B member 1 (SCARB1), and PPARG [23–25].

Therefore, the purpose of this study was to evaluate the causal effect of lipid-regulating targets on DR outcome using a two-sample MR study. To our knowledge, it is the first study investigating the association between lipid profiles and DR risk through specific lipid-regulating targets. Findings from this study suggest that lipid-lowering therapies may reduce the risk of DR. Furthermore, it provides insights into specific therapeutic targets through which such therapies could potentially reduce DR risk. These results underscore the importance of integrated disease management for protecting visual health in people with diabetes.

## 2. Materials and Methods

### 2.1. Study Design

This study was a two-sample MR analysis based on publicly available GWAS summary-level data. The present study incorporated eight lipid-regulating targets, comprising three LDL-C targets (HMGCR, PCSK9, and NPC1L1), along with three HDL-C targets (CETP, SCARB1, and PPARG) and two TG targets (PPARA and LPL) as exposures. The outcome of the present study was DR.

No further ethical approval was necessary as the data were obtained from publicly available databases.

### 2.2. Data Source

Genetic instruments for blood LDL-C (GWAS ID: ieu-a-300) [26], HDL-C (GWAS ID: ieu-b-109) [27], and TG (ieu-b-111 and ebi-a-GCST90092829) [27, 28] were obtained from the IEU OpenGWAS project [29]. GWAS summary-level data for DR (scale of 0 to 4 including no DR, mild, moderate, severe, and proliferative DR [30]) was obtained from finn-b-H7_RETINOPATHYDIAB_BKG in FinnGen data release 9. Detailed information for each dataset can be seen in Supplementary Table 1 (details of the GWAS datasets).

### 2.3. Selection of Genetic Instruments

Single nucleotide polymorphisms (SNPs) located within 100 kb windows of the target genes (HMGCR, PCSK9, and NPC1L1) and exhibiting a genome-wide significance level association (*P* < 5.0 × 10^−8^) with LDL-C were chosen as proxies for the targets related to LDL-C. In a similar manner, SNPs located within 100 kb windows of CETP, SCARB1, and PPARG, which demonstrated significant associations with HDL-C at the genome-wide level, were employed as proxies for the targets associated with HDL-C. And SNPs, located within 100 kb windows of PPARA and LPL which demonstrated significant associations with TG at the genome-wide level, were employed as TG targets. SNPs with a linkage disequilibrium coefficient (*r*^2^ < 0.10) and an effective allele frequency > 0.01 were selected as independent IVs to perform MR analysis.

### 2.4. Statistical Analyses

Inverse-variance-weighted MR (IVW-MR) was used to calculate the effect estimates by each target. If heterogeneity exists, random-effects IVW model is applied; otherwise, the fixed-effects IVW model is applied [31, 32]. The odds ratio (OR) and 95% confidence interval (CI) values were calculated. Sensitivity analyses including heterogeneity test, horizontal pleiotropy test, and leave-one-out method have been described in detail in our previous study [33, 34]. All analyses were performed using the TwoSampleMR R package (version 0.5.7). The Bonferroni correction was used to adjust the thresholds of significance level. Strong evidence of significance was suggested for *P* values less than 0.00625 (adjusted for eight comparisons), and there was suggestive evidence for *P* values ranging from 0.00625 to less than 0.05 [35].

## 3. Results

### 3.1. Acquisition of Genetic Instruments for Drug Targets

Following the screening criteria, a total of 2 SNPs located within or in close proximity (100 kb) to HMGCR were chosen as IVs for the LDL-C target to conduct MR analysis. Similarly, 10 SNPs for PCSK9 and 2 SNPs for NPC1L1 were selected. Additionally, 53, 18, and 5 SNPs were selected as IVs for the HDL-C targets CETP, SCARB1, and PPARG, respectively. Lastly, 2 SNPs for PPARA and 11 SNPs for LPL were selected as IVs for the TG targets. IVs ultimately used for MR analysis can be found in Supplementary materials.

Supplementary Tables 2–9 show the IVs used to assess the genetic association between HMGCR, PCSK9, NPC1L1, CETP, SCARB1, PPARG, PPARA, and LPL targets and DR, respectively.

### 3.2. Association between Drug Targets and DR

**Figure 1:**
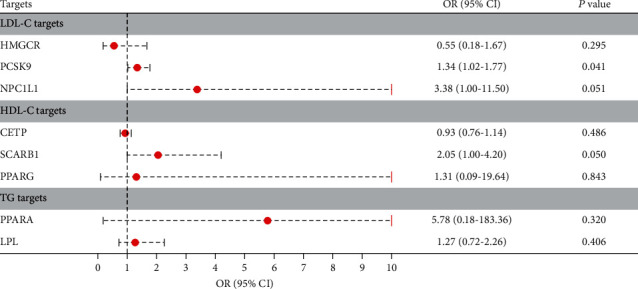
Association between lipid-regulating drug targets and DR risk.

**Figure 2:**
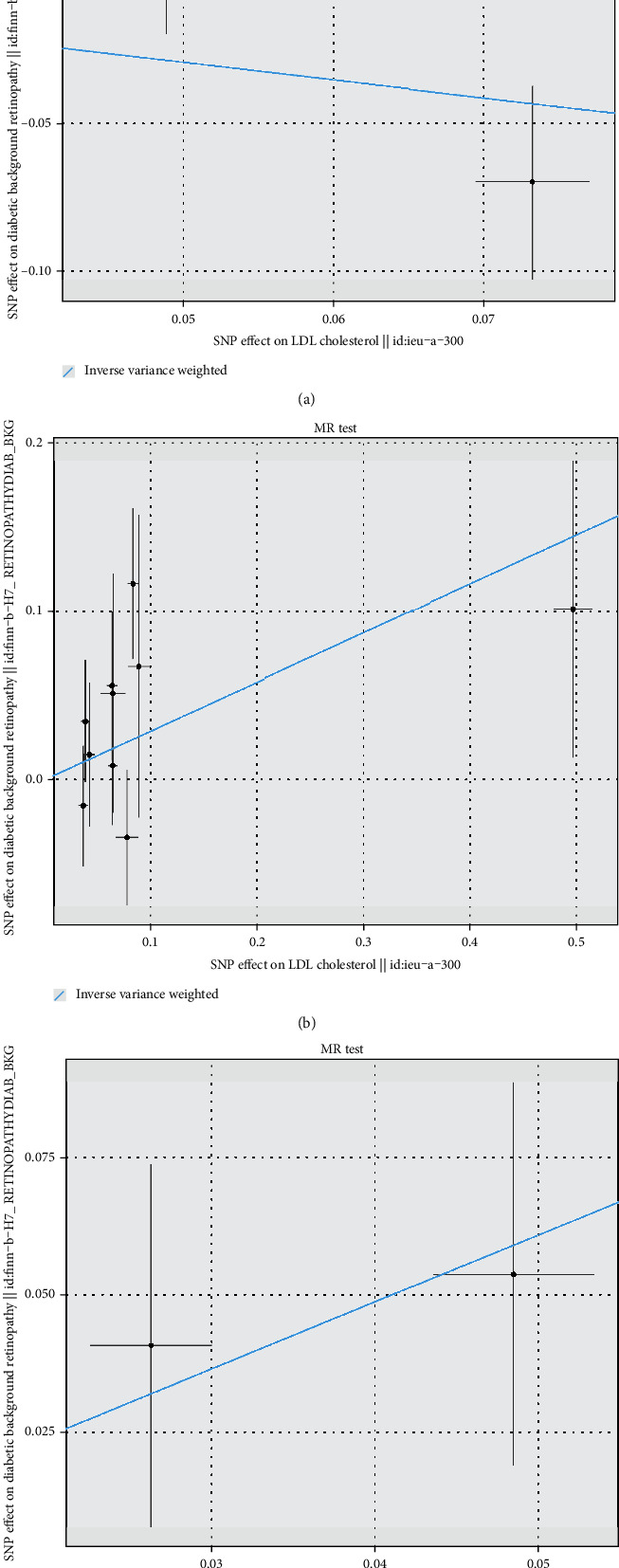
Scatter plot for drug targets: (a) HMGCR-, (b) PCSK9-, and (c) NPC1L1-mediated LDL-C effects on DR.

**Figure 3:**
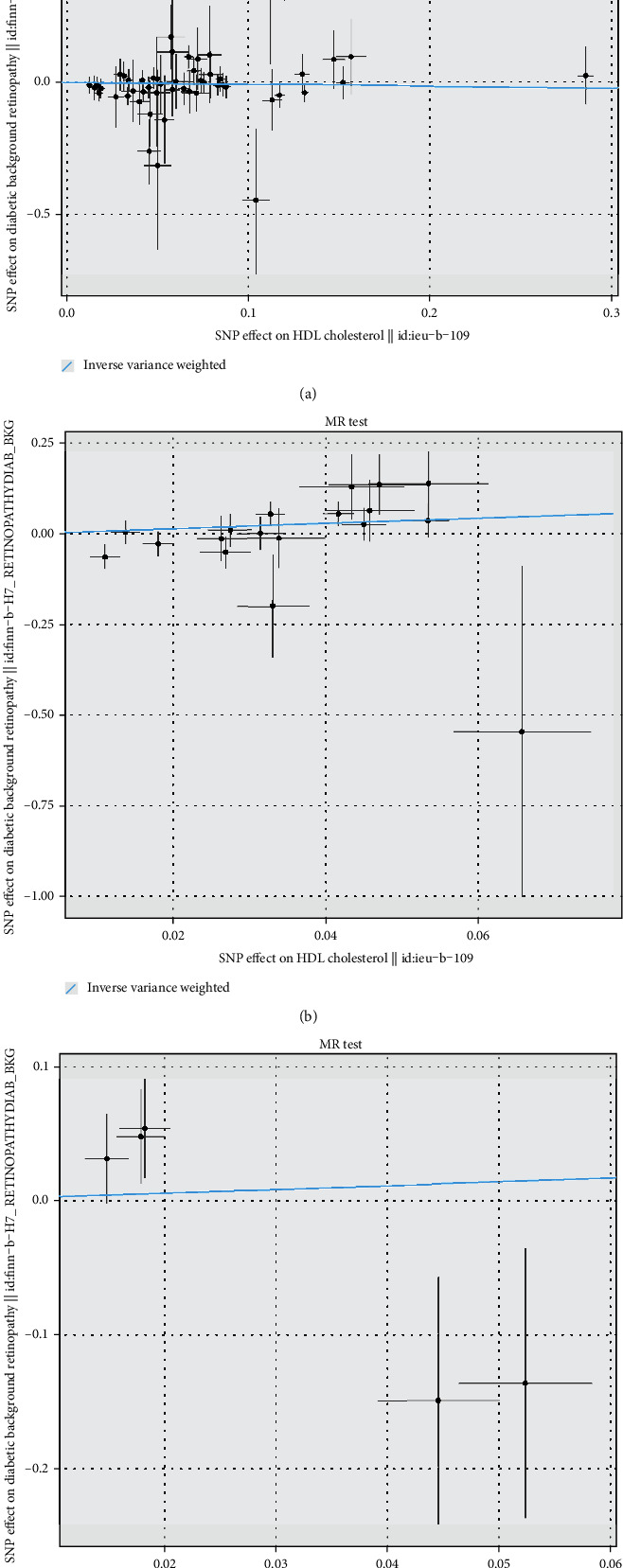
Scatter plot for drug targets: (a) CETP-, (b) SCARB1-, and (c) PPARG-mediated HDL-C effects on DR.

**Figure 4:**
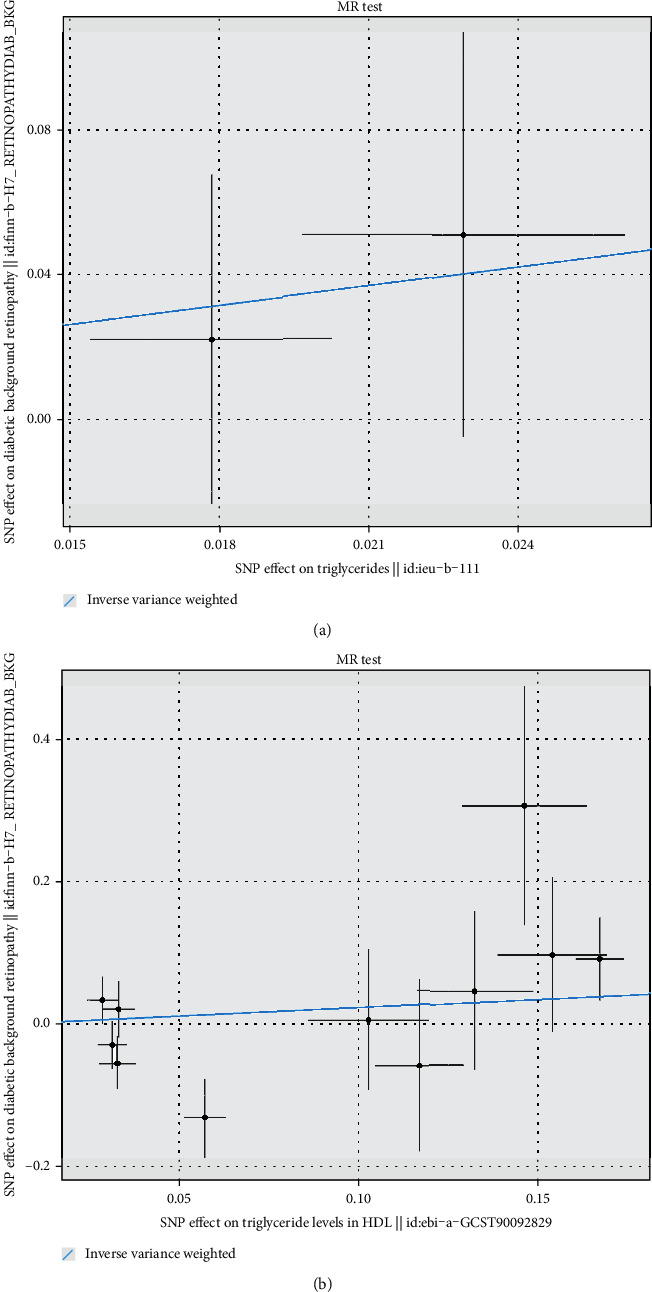
Scatter plot for drug targets: (a) PPARA- and (b) LPL-mediated TG effects on DR.

IVW-MR was used to assess the association between drug targets and DR. The results provided suggestive evidence of a positive association between PCSK9-mediated LDL-C levels and the risk of DR, with an OR and 95% CI of 1.34 (1.02-1.77) (Figures 1 and 2(b)). No significant association was observed between the HMGCR- and NPC1L1-mediated LDL-C levels (Figures 1 and 2); CETP-, SCARB1-, and PPARG-mediated HDL-C levels (Figures 1 and 3); and PPARA- and LPL-mediated TG levels (Figures 1 and 4) with the risk of DR.

### 3.3. Sensitivity Analyses

Based on Cochran's *Q* test, no significant heterogeneity was observed across all targets (Table 1). Pleiotropy test for HMGCR, NPC1L1, and PPARA targets could not be conducted as insufficient genetic instruments were identified. No horizontal pleiotropy was observed in other targets in MR-PRESSO global test (Table 2). Horizontal pleiotropy was detected in SCARB1 targets in MR-Egger regression.

## 4. Discussion

This MR study provides suggestive evidence of a positive association between PCSK9-mediated LDL-C levels and DR risk at the genetic level. These findings suggest that inhibitors of PCSK9 may potentially provide protective effects against DR. To the best of our knowledge, this is the first study to establish a causal relationship between lipid-regulating drug targets and DR risk at the genetic level. The absence of horizontal pleiotropy and heterogeneity in the PCSK9 target underlines the robustness of the result.

In contrast to previous studies that focused only on the relationship between lipid levels and DR risk, we investigated the effect of specific lipid-regulating targets on DR. Among the eight targets analyzed in this study, we found that elevated PCSK9-mediated LDL-C levels may be associated with an increased risk of DR. The results of our study allow us to hypothesize that there is a causal relationship between PCSK9 inhibition and the reduced risk of DR. However, no evidence was found regarding the association between DR risk and other LDL-C-associated targets including HMGCR and NPC1L1. Meanwhile, none of the HDL-C and TG targets were related to DR risk. Thus, the results of this study indicate that modulation of the LDL-C target, rather than the TG or HDL-C target, may reduce the risk of DR. Moreover, it is likely that PCSK9 mediate this effect. Therefore, in addition to lipid-lowering effects, the use of PCSK9 inhibitors may potentially contribute to the reduction of retinal diseases in diabetic patients.

PCSK9 is a critical lipid metabolism gene that targets LDL receptors on the surface of liver cells [36]. PCSK9 inhibitors can increase the abundance of the LDL receptors to reduce LDL-C levels [37]. Some studies found significantly higher PCSK9 levels in patients with diabetes compared to those without diabetes [38–41]. Recent evidence from a meta-analysis, which included eight large randomized controlled trials, showed that PCSK9 inhibitors effectively reduced the risk of major cardiovascular events (MACE) and improved lipid profiles in patients with diabetes and dyslipidaemia over a median follow-up of 51 weeks [42]. Therefore, although PCSK9 inhibitors may mildly elevate blood glucose [43, 44], their long-term benefits would outweigh this potential risk [45, 46]. Our study further provides evidence for the potential of PCSK9 inhibition in mitigating the risk of DR at the genetic level.

Furthermore, our study yielded reliable conclusions indicating that TG levels mediated by LPL and HDL-C levels mediated by CETP and PPARG are not associated with the risk of DR. However, it is noteworthy that horizontal pleiotropy was detected in the HDL-C targets SCARB1 in MR-Egger regression. IVW estimates would be biased if horizontal pleiotropy existed [47], because the existence of horizontal pleiotropy can induce false-positive causal relationships in up to 10% of relationships [48]. Since no horizontal pleiotropy was observed in these targets by MR-PRESSO global test and negative results were obtained in the current analysis, we do not consider the effects caused by horizontal pleiotropy. Despite our efforts to utilize various datasets, we were unable to acquire an adequate number of genetic instruments, thus preventing us from conducting a pleiotropy analysis on the targets HMGCR, NPC1L1, and PPARA. Meanwhile, the LDL-C dataset includes a minor fraction of non-European ancestry, accounting for 4.02% of the sample. Although the original study has shown that population stratification has a negligible effect on its results [26], it remains crucial to consider that the presence of different ancestries may still influence SNP weights [49]. Therefore, to further validate these findings and enhance their generalizability, it is essential to conduct genomic studies with larger sample sizes across independently represented ethnicities.

Although the evidence is still preliminary, these results suggest that the use of PCSK9 inhibitors may help to reduce the risk of DR. The results of our study also provide some evidence for a better selection of the most appropriate lipid-regulating drugs in patients with diabetes. Further evidence from experimental and real-world clinical studies is necessary to confirm these findings.

The present study has the following advantages compared to previous researches. This is the first MR study to assess the genetic association between lipid-regulating targets, including LDL-C, HDL-C, TG, and DR risk. The application of genetic instruments to proxy drug target exposure could minimize confounding bias and avoid reverse causation. We focused the effects of lipid-regulating targets on DR, which would not only help analyze the relationship between lipid profiles and DR but also provide evidence for identifying precise therapeutic targets. The large sample size in GWAS datasets allows us to draw the above conclusions.

Our study also has several limitations. First, the present study was based on GWAS summary data but not individual level, and we could not conduct subgroup analyses according to the severity of DR. Second, targets included in this study are limited to known important drug targets, which cannot represent all potential targets. The GWAS datasets used in the present analysis were predominantly derived from the European population, which may limit the generalizability of the findings to other populations. Therefore, future studies that stratify disease severity across different genetic ancestries are needed to fully elucidate the relationship between lipid-regulating drug targets and DR and translate these findings into clinically actionable insights.

## 5. Conclusions

This study provides suggestive evidence of a positive association between PCSK9-mediated LDL-C levels and DR risk at the genetic level. PCSK9 could be potential prognostic biomarkers and therapeutic targets for DR. PCSK9 inhibition may reduce the risk of DR.

## Figures and Tables

**Table 1:** The results of heterogeneity test.

Exposure	IVW Cochran's *Q* test	
	*Q* value	*P* value
LDL-C targets		
HMGCR	2.250111	0.134
PCSK9	8.657546	0.469
NPC1L1	0.093398	0.760
HDL-C targets		
CETP	35.903940	0.957
SCARB1	18.554610	0.355
PPARG	9.182818	0.057
TG targets		
PPARA	0.076553	0.782
LPL	15.867860	0.103

**Table 2:** The results of pleiotropy test.

Exposure	Intercept in MR-Egger regression		RSSobs in MR-PRESSO global test	
	Value	*P* value	Value	*P* value
LDL-C targets				
HMGCR	—	—	—	—
PCSK9	0.010891	0.602	11.455660	0.505
NPC1L1	—	—	—	—
HDL-C targets				
CETP	-0.015321	0.268	36.964580	0.968
SCARB1	-0.068692	0.026	19.968930	0.388
PPARG	0.133891	0.065	14.509800	0.073
TG targets				
PPARA	—	—	—	—
LPL	-0.038611	0.227	19.424070	0.153

## Data Availability

The datasets are available in the IEU OpenGWAS and FinnGen research project.

## Supplementary Materials

Supplementary 1 Table 1: details of the GWAS datasets.

Supplementary 2 Table 2: genetic instruments used to assess the genetic association between HMGCR target and DR.

Supplementary 3 Table 3: genetic instruments used to assess the genetic association between PCSK9 target and DR.

Supplementary 4 Table 4: genetic instruments used to assess the genetic association between NPC1L1 target and DR.

Supplementary 5 Table 5: genetic instruments used to assess the genetic association between CETP target and DR.

Supplementary 6 Table 6: genetic instruments used to assess the genetic association between SCARB1 target and DR.

Supplementary 7 Table 7: genetic instruments used to assess the genetic association between PPARG target and DR.

Supplementary 8 Table 8: genetic instruments used to assess the genetic association between PPARA target and DR.

Supplementary 9 Table 9: genetic instruments used to assess the genetic association between LPL target and DR.

## References

[1] Hou X., Wang L., Zhu D. (2023). Prevalence of diabetic retinopathy and vision-threatening diabetic retinopathy in adults with diabetes in China. *Nature Communications*.

[2] Teo Z. L., Tham Y. C., Yu M. (2021). Global prevalence of diabetic retinopathy and projection of burden through 2045: systematic review and meta-analysis. *Ophthalmology*.

[3] Cheung N., Mitchell P. (2010). Diabetic retinopathy. *Lancet*.

[4] Yan S., Meng Z., Tian S. (2020). Neonicotinoid insecticides exposure cause amino acid metabolism disorders, lipid accumulation and oxidative stress in ICR mice. *Chemosphere*.

[5] Pickering R. J., Rosado C. J., Sharma A., Buksh S., Tate M., De Haan J. B. (2018). Recent novel approaches to limit oxidative stress and inflammation in diabetic complications. *Clinical & Translational Immunology*.

[6] Antonetti D. A., Silva P. S., Stitt A. W. (2021). Current understanding of the molecular and cellular pathology of diabetic retinopathy. *Nature Reviews Endocrinology*.

[7] Li N., Zhang X., Zhang M. (2023). Associations of genetically determined lipid traits and lipid-modifying agents with the risk of diabetic retinopathy: a Mendelian randomization study. *Atherosclerosis*.

[8] Zhang Z., Zhao L., Zhou X., Meng X., Zhou X. (2022). Role of inflammation, immunity, and oxidative stress in hypertension: new insights and potential therapeutic targets. *Frontiers in Immunology*.

[9] Choi L. S., Ahmed K., Kim Y. S., Yim J. E. (2022). Skin accumulation of advanced glycation end products and cardiovascular risk in Korean patients with type 2 diabetes mellitus. *Heliyon*.

[10] Bryl A., Mrugacz M., Falkowski M., Zorena K. (2022). The effect of diet and lifestyle on the course of diabetic retinopathy-a review of the literature. *Nutrients*.

[11] Hirai F. E., Moss S. E., Klein B. E., Klein R. (2008). Relationship of glycemic control, exogenous insulin, and C-peptide levels to ischemic heart disease mortality over a 16-year period in people with older-onset diabetes: the Wisconsin epidemiologic study of diabetic retinopathy (WESDR). *Diabetes Care*.

[12] Morton J., Zoungas S., Li Q. (2012). Low HDL cholesterol and the risk of diabetic nephropathy and retinopathy: results of the ADVANCE study. *Diabetes Care*.

[13] Jeng C. J., Hsieh Y. T., Yang C. M., Yang C. H., Lin C. L., Wang I. J. (2018). Diabetic retinopathy in patients with dyslipidemia: development and progression. *Ophthalmology Retina*.

[14] Li Z., Yuan Y., Qi Q., Wang Q., Feng L. (2023). Relationship between dyslipidemia and diabetic retinopathy in patients with type 2 diabetes mellitus: a systematic review and meta-analysis. *Systematic Reviews*.

[15] Yuan J., Xiong X., Zhang B. (2022). Genetically predicted C-reactive protein mediates the association between rheumatoid arthritis and atlantoaxial subluxation. *Frontiers in Endocrinology*.

[16] Saadh M. J., Pal R. S., Arias-Gonzáles J. L. (2023). A Mendelian randomization analysis investigates causal associations between inflammatory bowel diseases and variable risk factors. *Nutrients*.

[17] Howe L. J., Tudball M., Davey Smith G., Davies N. M. (2022). Interpreting Mendelian-randomization estimates of the effects of categorical exposures such as disease status and educational attainment. *International Journal of Epidemiology*.

[18] Levin M. G., Klarin D., Assimes T. L. (2021). Genetics of smoking and risk of atherosclerotic cardiovascular diseases: a Mendelian randomization study. *JAMA Network Open*.

[19] Sobrin L., Chong Y. H., Fan Q. (2017). Genetically determined plasma lipid levels and risk of diabetic retinopathy: a Mendelian randomization study. *Diabetes*.

[20] Jenkins A. J., Grant M. B., Busik J. V. (2022). Lipids, hyperreflective crystalline deposits and diabetic retinopathy: potential systemic and retinal-specific effect of lipid-lowering therapies. *Diabetologia*.

[21] Wolska A., Lo L., Sviridov D. O. (2020). A dual apolipoprotein C-II mimetic-apolipoprotein C-III antagonist peptide lowers plasma triglycerides. *Science Translational Medicine*.

[22] Lin C. Y., Chen P. Y., Hsu H. J., Gao W. Y., Wu M. J., Yen J. H. (2022). The citrus flavonoid nobiletin downregulates angiopoietin-like protein 3 (ANGPTL3) expression and exhibits lipid-modulating effects in hepatic cells and adult zebrafish models. *International Journal of Molecular Sciences*.

[23] Amaya-Montoya M., Pinzón-Cortés J. A., Silva-Bermúdez L. S. (2020). ApoE and apoC-III-defined HDL subtypes: a descriptive study of their lecithin cholesterol acyl transferase and cholesteryl ester transfer protein content and activity. *Lipids in Health and Disease*.

[24] Acton S., Rigotti A., Landschulz K. T., Xu S., Hobbs H. H., Krieger M. (1996). Identification of scavenger receptor SR-BI as a high density lipoprotein receptor. *Science*.

[25] Engwa G. A., Nwalo F. N., Chiezey V. O., Unachukwu M. N., Ojo O. O., Ubi B. E. (2018). Assessment of the Pro12Ala polymorphism in the *PPAR-γ2* gene among type 2 diabetes patients in a Nigerian population. *Journal of Clinical Medicine*.

[26] Willer C. J., Schmidt E. M., Sengupta S. (2013). Discovery and refinement of loci associated with lipid levels. *Nature Genetics*.

[27] Richardson T. G., Sanderson E., Palmer T. M. (2020). Evaluating the relationship between circulating lipoprotein lipids and apolipoproteins with risk of coronary heart disease: a multivariable Mendelian randomisation analysis. *PLoS Medicine*.

[28] Richardson T. G., Leyden G. M., Wang Q. (2022). Characterising metabolomic signatures of lipid-modifying therapies through drug target Mendelian randomisation. *PLoS Biology*.

[29] Elsworth B., Lyon M., Alexander T. (2020). *The MRC IEU OpenGWAS data infrastructure*.

[30] Emma Dugas J., Jorge W. C. (2015). *Diabetic retinopathy detection*.

[31] Yuan S., Kim J. H., Xu P., Wang Z. (2022). Causal association between celiac disease and inflammatory bowel disease: a two-sample bidirectional Mendelian randomization study. *Frontiers in Immunology*.

[32] Papadimitriou N., Dimou N., Tsilidis K. K. (2020). Physical activity and risks of breast and colorectal cancer: a Mendelian randomisation analysis. *Nature Communications*.

[33] Chen S., Zhang W., Zheng Z. (2023). Unraveling genetic causality between type 2 diabetes and pulmonary tuberculosis on the basis of Mendelian randomization. *Diabetology and Metabolic Syndrome*.

[34] Chen S., Zhang M., Zhang W. (2024). The causal association between blood Lead and sleep disorders: Evidence from National Health and nutrition examination survey and Mendelian randomization analysis. *Journal of Epidemiology and Global Health*.

[35] Huang W., Xiao J., Ji J., Chen L. (2021). Association of lipid-lowering drugs with COVID-19 outcomes from a Mendelian randomization study. *eLife*.

[36] Xu Q., Zhao Y. M., He N. Q. (2023). PCSK9: a emerging participant in heart failure. *Biomedicine & Pharmacotherapy*.

[37] Zhu Z., Zhang F., Hu H. (2016). Integration of summary data from GWAS and eQTL studies predicts complex trait gene targets. *Nature Genetics*.

[38] Awadallah S., Taneera J., Mohammed A. K., Unnikannan H., Sulaiman N. (2020). Combined intake of glucose-and lipid-lowering medications further elevates plasma levels of PCSK9 in type 2 diabetes patients. *Diabetes and Metabolic Syndrome: Clinical Research and Reviews*.

[39] Guo W., Gong Y., Gu Y. (2018). Circulating PCSK9 levels and 2-hPG are positively correlated in metabolic diseases in a Chinese Han population. *Lipids in Health and Disease*.

[40] Wu Y., Shi J., Su Q., Yang Z., Qin L. (2022). Correlation between circulating PCSK9 levels and gestational diabetes mellitus in a Chinese population. *Frontiers in Endocrinology*.

[41] Bojanin D., Vekic J., Milenkovic T. (2019). Association between proprotein convertase subtilisin/kexin 9 (PCSK9) and lipoprotein subclasses in children with type 1 diabetes mellitus: effects of glycemic control. *Atherosclerosis*.

[42] Imbalzano E., Ilardi F., Orlando L., Pintaudi B., Savarese G., Rosano G. (2023). The efficacy of PCSK9 inhibitors on major cardiovascular events and lipid profile in patients with diabetes: a systematic review and meta-analysis of randomized controlled trials. *European Heart Journal - Cardiovascular Pharmacotherapy*.

[43] Da Dalt L., Ruscica M., Bonacina F. (2019). PCSK9 deficiency reduces insulin secretion and promotes glucose intolerance: the role of the low-density lipoprotein receptor. *European Heart Journal*.

[44] Goldman A., Raschi E., Cukierman-Yaffe T. (2022). Hyperglycaemic disorders associated with PCSK9 inhibitors: a real-world, pharmacovigilance study. *European Journal of Preventive Cardiology*.

[45] D'Onofrio N., Prattichizzo F., Marfella R. (2023). SIRT3 mediates the effects of PCSK9 inhibitors on inflammation, autophagy, and oxidative stress in endothelial cells. *Theranostics*.

[46] Sabatine M. S., Leiter L. A., Wiviott S. D. (2017). Cardiovascular safety and efficacy of the PCSK9 inhibitor evolocumab in patients with and without diabetes and the effect of evolocumab on glycaemia and risk of new-onset diabetes: a prespecified analysis of the FOURIER randomised controlled trial. *The Lancet Diabetes and Endocrinology*.

[47] Chen X., Hong X., Gao W. (2022). Causal relationship between physical activity, leisure sedentary behaviors and COVID-19 risk: a Mendelian randomization study. *Journal of Translational Medicine*.

[48] Verbanck M., Chen C. Y., Neale B., Do R. (2018). Detection of widespread horizontal pleiotropy in causal relationships inferred from Mendelian randomization between complex traits and diseases. *Nature Genetics*.

[49] Mosley J. D., Gupta D. K., Tan J. (2020). Predictive accuracy of a polygenic risk score compared with a clinical risk score for incident coronary heart disease. *Journal of the American Medical Association*.

